# Orchard recycling improves climate change adaptation and mitigation potential of almond production systems

**DOI:** 10.1371/journal.pone.0229588

**Published:** 2020-03-27

**Authors:** Emad Jahanzad, Brent A. Holtz, Cameron A. Zuber, David Doll, Kelsey M. Brewer, Sean Hogan, Amélie C. M. Gaudin

**Affiliations:** 1 Department of Plant Sciences, University of California, Davis, California, United States of America; 2 University of California Agriculture and Natural Recourses, Davis, California, United States of America; Universidade de Coimbra, PORTUGAL

## Abstract

There is an urgent need to develop climate smart agroecosystems capable of mitigating climate change and adapting to its effects. In California, high commodity prices and increased frequency of drought have encouraged orchard turnover, providing an opportunity to recycle tree biomass *in situ* prior to replanting an orchard. Whole orchard recycling (WOR) has potential as a carbon (C) negative cultural practice to build soil C storage, soil health, and orchard productivity. We tested the potential of this practice for long term C sequestration and hypothesized that associated co-benefits to soil health will enhance sustainability and resiliency of almond orchards to water-deficit conditions. We measured soil health metrics and productivity of an almond orchard following grinding and incorporation of woody biomass vs. burning of old orchard biomass 9 years after implementation. We also conducted a deficit irrigation trial with control and deficit irrigation (-20%) treatments to quantify shifts in tree water status and resilience. Biomass recycling led to higher yields and substantial improvement in soil functioning, including nutrient content, aggregation, porosity, and water retention. This practice also sequestered significantly higher levels of C in the topsoil (+5 t ha^-1^) compared to burning. We measured a 20% increase in irrigation water use efficiency and improved soil and tree water status under stress, suggesting that *in situ* biomass recycling can be considered as a climate smart practice in California irrigated almond systems.

## Introduction

The major global challenge facing agriculture is to increase sustainable food production in a changing climate while simultaneously reducing greenhouse gas emissions. Enhancing soil carbon (C) content of agricultural soils provides enormous potential to help reach these goals as C sequestration mitigates climate change [1,2] but also builds sustainability and resilience by improving plant growth conditions and conservation of natural resources [3,4]. Soil organic C (SOC) is essential for soils to function as living ecosystems that provide and cycle nutrients, protect crops from pests and pathogens, conserve water, and reduce risks of soil erosion, thus minimizing the environmental footprint of agriculture while increasing adaptation to harsh climatic conditions [5]. Ambitious climate mitigation and adaptation policies are increasingly recognizing this potential and incentivizing the development and adoption of innovative management practices that promote cycling and storage of SOC. However, uncertainties remain about the potential of management practices to help meet regional climate smart agriculture and soil health targets. Given the anticipated larger incentives to offset CO_2_ emissions by investing in increasing agricultural soil C storage, it is essential to estimate the potential for C gains and soil health co-benefits associated with specific management practices as well as the downstream impacts on adaptation to future climate conditions.

Biomass recycling is gaining widespread interest as a means to use agriculture and its by-products as a resource for renewable energy production. Grinding and incorporating whole trees into soil during orchard turnover may also provide a unique opportunity to store C in soils and build synergies between mitigation and adaptation goals at large scales [6,7]. This may be of particular significance for drought-prone semi-arid agroecosystems with lower levels of SOC and water retention, and with high vulnerability to impending shifts in resource availability [8]. Perennial cropping systems, such as California almonds [*Prunus dulcis* (Mill.) D. A. Webb], usually have low SOC and are uniquely susceptible to the impacts of a changing climate, including increased frequency of extreme weather events, constrained water resources, and reduced winter chill hours [9]. These production systems are prominent across the landscape and rapidly increasing with more than 530,000 hectares supplying 80% of global almond demand [10]. High commodity prices, costly and increasingly scarce water inputs, and more stringent air quality regulations have promoted turnover of less productive orchards to new almond plantings and renewed interest in using *in situ* biomass recycling as a climate adaptation and mitigation tool [11,12]. However, the potential for long-term C sequestration and practical tradeoffs associated with high C input practices such as whole orchard recycling (WOR) remain unclear in semi-arid orchard production systems where production relies heavily on irrigation.

We tested the long-term viability of biomass recycling within test plots of a highly productive almond orchard and evaluated its potential benefits for climate change adaptation. We hypothesized that addition of substantial amounts of high organic C woody biomass would lead to long term soil C sequestration and improvements in soil health characteristics with positive impacts on yields and orchard productivity. We also explored the implications for orchard adaptation to future climate conditions by testing how and to what extent potential improvements in soil quality, especially hydraulic properties, would impact tree and yield resistance to an acute water shortage (i.e. deficit irrigation). Although it is often claimed that healthy soils have the potential to build resilience, this claim has seldom been empirically tested, especially in intensive perennial systems. This work provides novel insights on mitigation potential of this practice and the soil health mediated mechanisms influencing orchard health and productivity.

## Materials and methods

### Trial establishment and experimental design

This experiment was conducted in an irrigated almond orchard located at the University of California Kearney Agricultural Research and Extension Center in Parlier, CA (36°35'59.4"N 119°30'11.7"W) on a Hanford fine sandy loam site. The climate is Mediterranean, with precipitation levels below evapotranspiration (ET) requirements during most of the growing season. Long term (68 years) annual rainfall and temperature averages are 285 mm and 17°C, respectively. The soil treatments were established in a complete randomized block design with seven replications in 2008 following termination of a 20-year old peach (*Prunus persica* Var. Fay Elberta) orchard. The estimated amount of woody biomass returned to the soil, excluding roots, was ~74 t ha^-1^ comprised of ~50% C. Two soil treatments were established in 2008: i) the grind treatment (WOR), which consisted of whole trees ground and shredded using a land clearing equipment (Ironwolf 700B Slasher, Nobe, OK, USA) and incorporated within the top 15 cm of soil in the tree row. The IronWolf pushed standing trees over in place and ground them on its first forward pass using a 3-meter wide × 1-meter high head. On the second pass, going in reverse, the IronWolf lowered the rotating head into the soil so that the ground trees were incorporated into soil to a depth of 15 cm. The orchard was disked twice (0–15 cm) before berms were re-created down the previous tree-rows with a Disc-Ridger. ii) the burn treatment was established by uprooting and burning the trees and incorporating ashes into the topsoil (0–15 cm) in the tree row. The orchard was replanted in January of 2009 with three almond varieties (Nonpareil, Butte, and Carmel) on Nemaguard rootstock. All measurements were performed on the Nonpareil variety rows because i) they have been historically more intensively measured and monitored over the last nine years and ii) Nonpareil is the most commercially relevant variety in California. Spacing between trees was 5.5 m × 6.7 m and one variety was planted per row. Each plot was composed of 18 trees arranged as 1 row of 6 trees per variety. One-tree buffer per row was established between the grind and burn treatments. Trees were irrigated daily to match potential evapotranspiration needs using micro sprinklers. Trees were fertilized with urea ammonium nitrate (UAN, 32-0-0) fertilizer at a rate of 100 kg N ha^-1^ via three fertigation events April through May.

### Deficit irrigation trial

In summer of 2017, a deficit irrigation experiment was implemented on 6 replicated plots on the Nonpareil variety. A split-plot arrangement with three replications was used with two irrigation treatments as main plot effects and grind and burn treatments as sub-plots across three replicates (n = 12 plots). Irrigation treatments consisted of regular (100% ET) and deficit (80% ET for 28 days) irrigation based on reference crop water use, and the almond crop coefficient (Kc) [13]. The deficit irrigation treatment lasted for 28 days and was cut off at the hull split to avoid hull rot in Nonpareil variety [14,15]. Weekly evapotranspiration values for the reference crop were obtained from the state of California Irrigation Management Information System (CIMIS) station located at the Kearney site and Kc values for mature almond trees were obtained from Sanden [16]. Trees were irrigated using Bowsmith Fan-Jet Micro sprinklers (Bowsmith Inc, Exeter, CA, USA) with a flow rate of 38 to 65 liters per hour placed in the tree row mid-way between trees. The deficit irrigation treatment (80% ET) was imposed using emitters with 20% less output (30 to 52 liter per hour). Pressure regulators (DG-5025, Hendrickson Bros, Corona, CA, USA) were installed before the water meters to maintain constant pressure within each row. The deficit irrigation treatment started on June 5, 2017 and was cut off on July 3, 2017 (hull split). The irrigation treatment was applied only to the middle Nonpareil variety row. The amount of irrigation water applied to each treatment row was measured with in-line water meters during the experiment. Irrigation water use efficiency (IWUE) (kg m^-3^) was calculated as kernel yield (kg ha^-1^) divided by the volume of irrigation water (m^3^ ha^-1^) [15].

### Soil sampling and processing

Soil samples were collected on the Nonpareil rows from the deficit irrigation trial in spring of 2017, 9 years after incorporation of woody biomass, using AMS regular soil augurs (AMS Inc, American Falls, ID, USA). All samples were collected from the berms between the trees to a depth of 0–15 cm, which corresponds to the woody biomass incorporation depth. Only the middle row trees (Nonpareil) were used for sampling and field measurements and trees at the beginning and at the end of each tree row were not sampled to avoid edge effects. Three root zone samples per plot were taken and composited (n = 12) on the same day to a depth of 0–15 cm for biological analysis. Field-moist soil was sieved to 2 mm and stored at 4°C prior to assays of microbial biomass and enzyme activities. For soil chemical properties, five soil cores per plot were sampled composited (n = 12) and dried in a forced-air oven at 50°C prior to analysis. Three separate undisturbed soil cores were also taken from each plot (n = 36, not composited) and stored at 4°C until processing for aggregate stability and fractionation tests. In addition, five soil cores were collected from each plot (n = 60) for bulk density (BD) measurements.

### Soil analysis

#### Chemical properties

Soils were analyzed in house at the University of California Division of Agriculture and Natural Resources Analytical Laboratory. The pH was determined using a saturated paste method [17]. Electrical conductivity (EC) was measured according to the method described by Rhoades [18]. Sodium (Na), Ca, Mg, K, B, S and micronutrients (Zn, Mn, Fe, Cu, and Mo) were measured using Inductively Coupled Plasma Emission Spectroscopy (ICP-AES) [19]. Cation exchange capacity (CEC) was determined based on the method described by Rible and Quick [20]. Soil nitrate, extractable ammonium, and Cl were measured using Flow Injection Analysis Colorimetry (QuikChem, Lachat Instruments, Milwaukee, WI, USA) [21]. Total N and C were determined using the combustion method (ECS 4010, Costech Analytical Technologies Inc, Valencia, CA; USA). Soil C stock was calculated as the product of soil C concentration, soil sample bulk density, and sampling depth [22–24]. Soil organic matter (SOM) was measured using the Loss-On-Ignition Method [25]. Permanganate Oxidizable C (POxC) was measured using the procedure described by Weil et al. [26].

#### Physical properties

Soil texture was determined using the hydrometer technique [27]. Soil compaction, defined as resistance, was measured using a soil cone penetrometer (Dickey-John, Auburn, IL, USA). Bulk density (BD) was measured according to Blake and Hartge [28]. Briefly, volume of soil (cm^3^) was calculated by subtracting volume of rocks from total soil volume. Volume of rocks was determined by submerging the rocks in a graduated cylinder with water and recording the difference before and after submerging (1 mL = 1 cm^-3^). The below equation was used to calculate BD:

Soil BD (g cm^-3^) = oven dry weight of soil / volume of soil

Soil water-stable aggregation and C and N content of different aggregate fractions were measured on undisturbed cores. Large particles were removed through sieving moist soil (8mm) and water-stable aggregates were separated by wet-sieving of a 40g subsample into four aggregate size fractions: >2000 μm (large macroaggregates), 250–2000 μm (small macroaggregates), 53–250 μm (microaggregates), and <53 μm (silt and clay fraction) according to a protocol modified from Elliot [29]. A vibratory sieve shaker with rainfall simulator (Fritsch Analysette 3 Pro, Pittsboro, NC, USA) was used. Aggregate fractions remaining on each sieve were oven-dried at 50°C and weighed. Carbon and N content of different aggregate fractions were measured using the combustion method. Mean weight diameter (MWD), a weighted-average index of aggregate stability, was calculated according to the following equation [30]:

$$MWD=\sum_{i=1}^{4}P_{i}*S_{i}$$

where *S*_*i*_ is the average diameter (μm) for particles in that fraction and *P*_*i*_ is the weight percentage of the fraction in the whole soil.

#### Hydraulic properties and water dynamics

Infiltration rate was obtained by measuring soil saturated hydraulic conductivity (Kfs) with a DualHead Infiltrometer (Decagon Devices, Inc., Pullman, WA, USA). Water‐retention characteristics of recycled orchard soils (grind) and control soils (burn) were determined using the evaporation method (HYPROP technique, 250-cm^3^ intact soil cores) (UMS HYPROP, Decagon Devices, Inc., Pullman, WA, USA) [31].

Soil water content was measured during the deficit irrigation trial at the 22, 45, 76, 106, and 137 cm depths using a neutron probe (CPN model 305 DR, Concord, CA, USA). One access tube was installed in each treatment plot next to a randomly selected Nonpareil trees (n = 12). The location of the access tubes was in-line with the tree row. To calibrate neutron probe readings, soil samples were collected from the same soil depths mentioned above upon installation of the access tubes to measure BD and then volumetric water content (%VWC). The actual moisture content of the soil at different depths were then calculated using the calibration curve from the %VWC of the samples and count ratio of slow neutrons (R^2^-value of 0.87 for all depths) [32]. Soil water content was measured between April 12 and July 24; measurements were made on a weekly basis during June 5 to July 3 and bi-weekly before and after the deficit irrigation experiment.

#### Biological properties

Microbial biomass C and N (MBC and MBN, respectively) were measured on field moist samples using the chloroform fumigation extraction method [33]. Carbon content in the extracts was determined using a TOC analyzer (TOC-V, Shimadzu, Japan). MBC was calculated as the difference between extractable organic C in unfumigated and fumigated samples, divided by a *Ke* factor of 0.35 [33]. Dissolved N in extracts was measured colorimetrically following the alkaline persulfate oxidation method [34]. MBN was calculated by dividing the difference in N content between the fumigated and unfumigated samples divided by 0.68 to account for incomplete N extraction [33].

The activity of various microbial enzymes involved in C and N cycling was measured in soil samples using fluorescence [35]. Targeted enzymes included: β-glucosidase (BG), β-D-cellobiosidase (CB), β-N-acetylglucosaminidase (NAG), and leucine aminopeptidase (LAP). Briefly, 2.75 g of field moist soil were homogenized with 91 mL of 50 mM buffer (NaOH) and stirred for one minute. 800 μL of soil slurry were used for each assay along with 200 μL of 4-Methylumbelliferone (MUB) and 7-Amino-4-methylcoumarin (MUC) of assay standards. 200 μL of BG, CB, NAG, and LAP substrates were added to the 96 well plates and then incubated in the dark at room temperature for three hours. Plates were then centrifuged for 3 minutes and fluorescence was read (Synergy^™^ H4 Hybrid Multi-Mode Microplate Reader, BioTek, Winooski, VT, USA).

#### Soil Health Index (SHI)

To calculate the SHI, samples were analyzed for water extractable organic C (WEOC), water extractable organic N (WEON), and 1-day CO_2_-C respiration based on a protocol described by Haney [36]. Briefly, total N was determined on 2 g dried subsamples using the combustion method. WEOC and water extractable N were measured on 4 g of soil in slurry after shaking for 10 minutes. Samples were centrifuged for 5 minutes at 3500 rpm, filtered through Whatman 2 V paper, and analyzed for WEOC (TOC analyzer) and water extractable N (Colorimetry). Inorganic NH_4_-N, and NO_3_-N concentrations were also determined using colorimetry technique [21]. WEON was calculated by subtracting inorganic N content (NH_4_-N and NO_3_-N) from water extractable N [37].

The flush of CO_2_ in 24 hours following rewetting of dried soil was measured on 40 g subsamples in 50 ml disposable beakers with four to five 6.35-mm holes drilled in the bottom fitted with a glass microfiber filter to prevent soil loss. The beaker and Solvita® gel paddle were placed in a gas-tight 250-mL glass jar filled with 25 mL of water and a convex bottom to allow for drainage. Capillary action was used to rewet soil samples, which were then incubated at 25°C, and respired CO_2_ was trapped over 24 h. The quantity of 1-day CO_2_-C released was determined using a digital-color reader (DCR) (www.solvita.com) [37]. These values were then used to calculate a SHI according to Haney [36]:

$$SHI=\frac{1-day\;CO2-C}{C:N+(WEOC-100+WEON-10)}$$

### Plant analysis

#### Yield and leaf analysis

Almond kernel yields (Nonpareil) were measured by mechanical harvest on September 2017. Gross and dry kernel weights (~3% moisture) were measured on subsamples from 6 trees per treatment plot. Twenty almond leaves per tree were sampled in mid-July, dried at 60°C, ground, sieved through 2-mm mesh and then sent to the University of California Division of Agriculture and Natural Resources (UC ANR) Analytical Laboratory for analysis of accumulated nutrients. Total Kjeldahl Nitrogen (TKN) was determined according to Horneck and Miller [38]. Phosphorus, K, S, Ca, Mg, Zn, Fe, Mn, Cu, B, Cl, Na, Al, and Mo were analyzed using nitric acid/hydrogen peroxide microwave digestion method and ICP-AES [19].

#### Tree water status

Tree water status was measured weekly using midday stem water potential (SWP) with a pressure chamber (Soilmoisture Equipment, Santa Barbara, CA, USA). Two lower-canopy, fully expanded, shaded leaves were measured after enclosing the leaves in bags made of plastic film and aluminum foil for at least one hour before measuring. Stomatal conductance (mmol m^-2^s^-1^) was measured on June 28 at solar noon using a leaf porometer (model SC-1, Decagon Devices, Inc., Pullman, WA, USA) on six randomly chosen leaves per tree in each plot (n = 72). Photosynthetically Active Radiations (PAR) intercepted by the tree canopy was measured as an indicator of tree growth using a utility vehicle at solar noon (model 610 Mule; Kawasaki Heavy Industries, Tokyo) fitted with a mobile PAR measurement platform [39] on August 25.

Aerial thermal images were acquired on June 28 in clear conditions between the hours of 12:00 and 13:00 to minimize effects of shadowing on the images. These images were collected at 70 meters above ground level with a metrically calibrated Zenmuse XT infrared thermal camera, mounted on a DJI Inspire 1 unmanned aerial vehicle. The Zenmuse thermal camera was equipped with a 9Hz FLIR microbolometer sensor, capturing 640 × 512 pixel frames (digital images) from a 640/30 fps from an active feed, with a sensitivity (NEdT) of 50 mK at f/1.0 in the spectral range of 7.5–13.5 μm, and a lens with an field of view of 69° × 56°. The aerial images were later processed in Pix4D Mapper Pro and ArcGIS software applications to produce a 9.2 cm spatial resolution orthomosaic map of the canopy temperature, segmenting each almond tree from surrounding soil pixels within the entire 2.37-acre orchard, to test whether there were significant temperature differences between the irrigation and burn vs. grind treatment areas.

### Statistical analysis

Data were analyzed using the PROC MIXED procedure of SAS [40]. Soil and irrigation treatments and the interaction of these factors were analyzed as fixed effects, and blocks and the interaction of blocks with main effects as random effects. The assumptions of ANOVA were tested, and transformations were applied where necessary to achieve normality of residuals. Weekly measurements of SWP and neutron probe readings were analyzed with repeated measure covariance structure [41]. When ANOVA showed significant differences (*P ≤ 0*.*05*), comparisons of means were made using the Tukey’s range test. Principal components analysis (PCA) was conducted using the JMP software [42]. Pearson correlation coefficients were calculated using the PROC CORR procedure to determine correlations between almond yield and soil health parameters as well as plant water status measurements [40]. The HYPROP data evaluation software (UMS GmbH) was used to fit the parameters of a model for the soil water retention q(h) and unsaturated hydraulic conductivity K(h) to the evaporation. We tested van Genuchten unimodal [43] and Durner bimodal retention model equations [44,45] and evaluated model fit quality with the corrected Akaike Information Criterion (AIC) [46]. Smaller AIC values indicate a better estimation quality of the model. Data were analyzed with the PROC MIXED model to compare soil treatments and means were separated using Tukey’s range test.

## Results

### Tree nutrition, yields, and productivity

**Fig 1:**
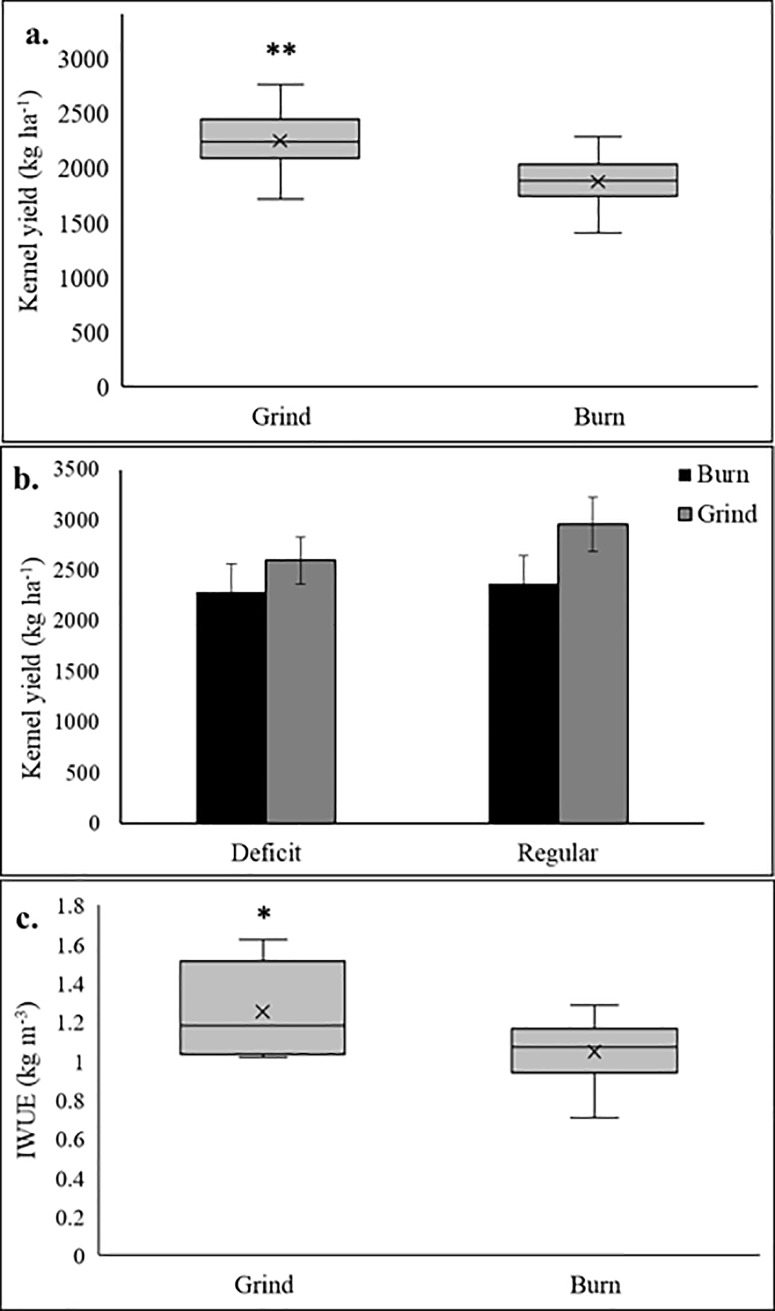
Effects of soil treatments (a) and soil treatments × irrigation regimes (b, *P = 0*.*25*) on kernel yields and irrigation water use efficiency (IWUE). (*) indicate significant difference at *P ≤0*.*05*.

Trees following WOR significantly out-yielded those in the burn treatment nine years after establishment (+19%, 400 kg ha^-1^; Fig 1A), which led to increased irrigation water use efficiency (IWUE) compared to the burn treatment (1.25 kg m^-3^ vs 1.04 kg m^-3^; Fig 1C). Higher kernel yields have been consistently observed in the grind treatment since 2014 (S1A Fig). Furthermore, the accumulated yields indicated a 15% kernel yield increase in the grind treatment compared to the burn (S1B Fig). Trees grown under WOR accumulated slightly higher leaf N content compared to the burning practice regardless of the irrigation treatments; yet, accumulation of other minerals was not significantly affected by the treatments (S1 Table).

### Soil carbon pools

**Fig 2:**
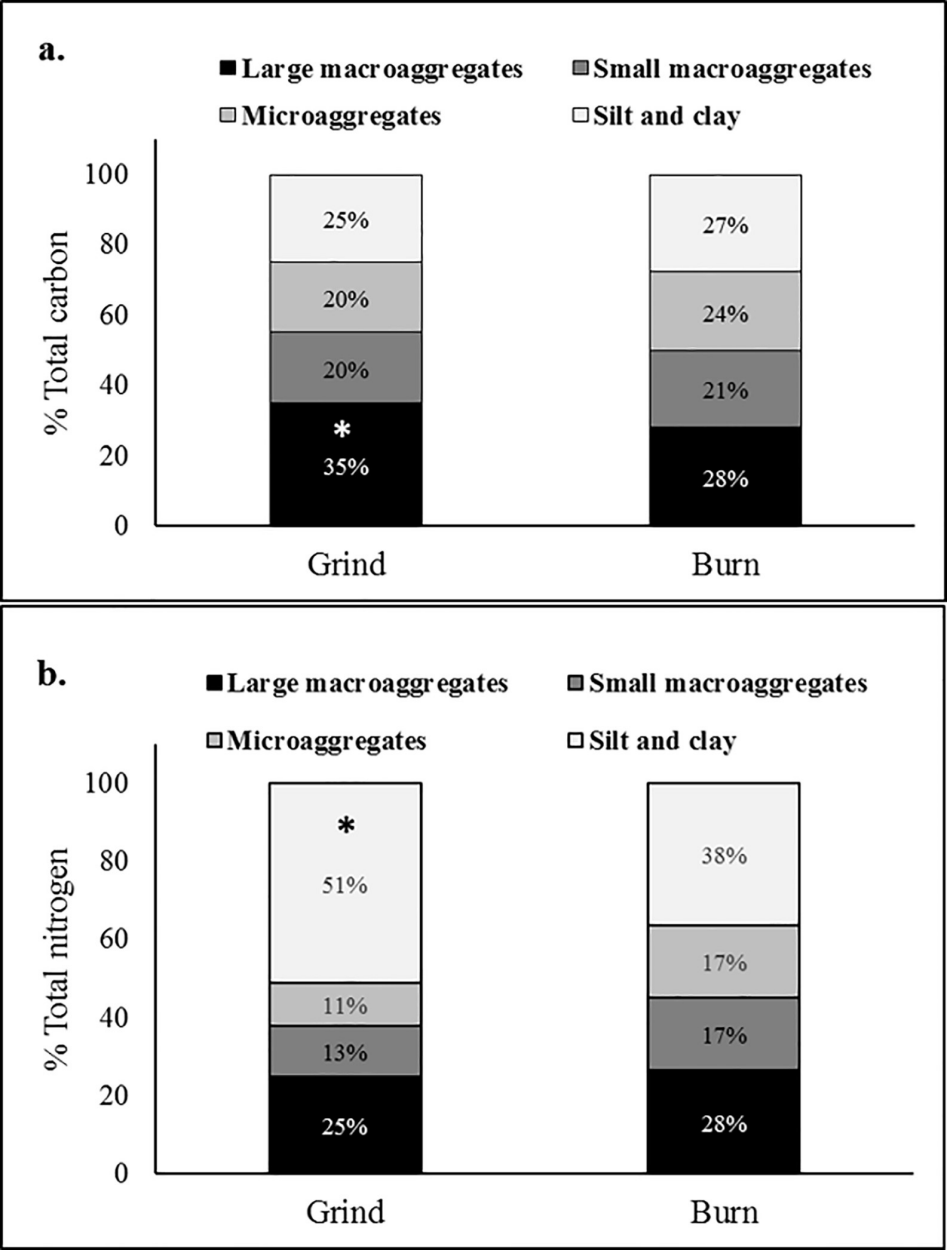
Impact of WOR on (a) total carbon and (b) nitrogen content in aggregate fractions. (*) indicate significant differences at *P ≤0*.*05*.

The grind treatment had higher SOM content (+ 0.45%) than the burn treatment nine years after WOR implementation (*P = 0*.*001;* Table 1). A proportional increase was observed in SOC of the grind compared to the burn treatment (Table 1). The total accumulation of soil C in the grind treatment was 5.2 tons per hectare (0–15 cm) higher than in the burn treatment (Table 1). Labile pools of SOC, such as POxC (Table 1), MBC (Table 2), and WEOC (Table 2), also increased relative to the burn treatment (+66, +47, and +26%, respectively). The distribution of C and N within aggregate fractions differed between the soil treatments, with a higher relative proportion and total weight of SOC occluded within large macroaggregates after grinding (35 vs 28% after burning, Fig 2A). Additionally, a larger proportion of TN was bound to minerals in the clay and silt fractions of the grind treatment (+ 34%) than in the burn treatment (Fig 2B).

**Table 1:** Impact of WOR on soil physical and chemical properties (0–15 cm) in 2017.

Treatment	BD (g cm^-3^)	Cpt (MPa)	EC (ds m^-1^)		CEC (meq^†^)		NO_3_^-^ (mg kg^-1^)	NH_4_^+^ (mg kg^-1^)	TN (%)	SOC (%)	C Stock (T ha^-1^)	SOM (%)	POxC (mg kg^-1^)
Grind	1.58	142	0.57		8.28		11.93	1.39	0.069	0.79	18.72	1.52	250
Burn	1.64	165	0.58		7.78		12.66	1.31	0.056	0.55	13.53	1.07	153
*P Value*	*0*.*05*	*0*.*05*	*0*.*45*		*0*.*28*		*0*.*73*	*0*.*35*	*0*.*05*	*0*.*001*	*0*.*01*	*0*.*001*	*0*.*04*
Treatment	pH	Ca	Mg	K		Na		Cl	B	Fe	Cu	Mn	Zn
		………………………………………………………….mg kg^-1^…………………………………………………………											
Grind	6.94	60.52	17.74	11.06		20.38		22.6	0.3	33.23	9.25	9.03	9.69
Burn	7.02	61.12	17.37	11.68		16.49		15.51	0.31	28.01	9.26	6.79	9.64
*P Value*	*0*.*39*	*0*.*48*	*0*.*47*	*0*.*39*		*0*.*03*		*0*.*003*	*0*.*4*	*0*.*11*	*0*.*5*	*0*.*01*	*0*.*47*

BD, bulk density; Cpt, compaction; EC, electrical conductivity; CEC, cation exchange capacity (^†^ Per 100 g of soil)

TN, total nitrogen; SOC, soil organic carbon; SOM, soil organic matter; POxC, permanganate oxidizable carbon

**Table 2:** Impact of WOR on microbial parameters and Soil Health Index determinants.

Treatment	MBN (μg N g soil^-1^)	MBC (μg C g soil^-1^)	Respiration (ppm)	WEON (ppm)	WEOC (ppm)	C:N	SHI
Grind	7.91	192	57.7	4.98	56.69	11.38	6.13
Burn	6.97	150	32.7	4.58	44.99	9.81	4.24
*P value*	*0*.*4113*	*0*.*0490*	*0*.*002*	*0*.*065*	*0*.*012*	*0*.*056*	*0*.*019*

MBN and MBC, Microbial biomass carbon and nitrogen, respectively; WEON, water extractable organic nitrogen; WEOC, water extractable organic carbon; C:N, carbon to nitrogen ratio of soil; SHI, soil health index.

### Soil chemical properties

Soil TN (+23%), Mn (+33%), Cl (+46%), and Na (+24%) contents were significantly greater following WOR implementation relative to the burn experiment (Table 1). Observed increases in TN content of the grind treatment did not result in increased NH_4_^+^ nor NO_3_^-^ contents. No significant differences were observed in other chemical properties, including pH, CEC, and EC (Table 1).

### Aggregation, compaction, and hydraulic properties

**Fig 3:**
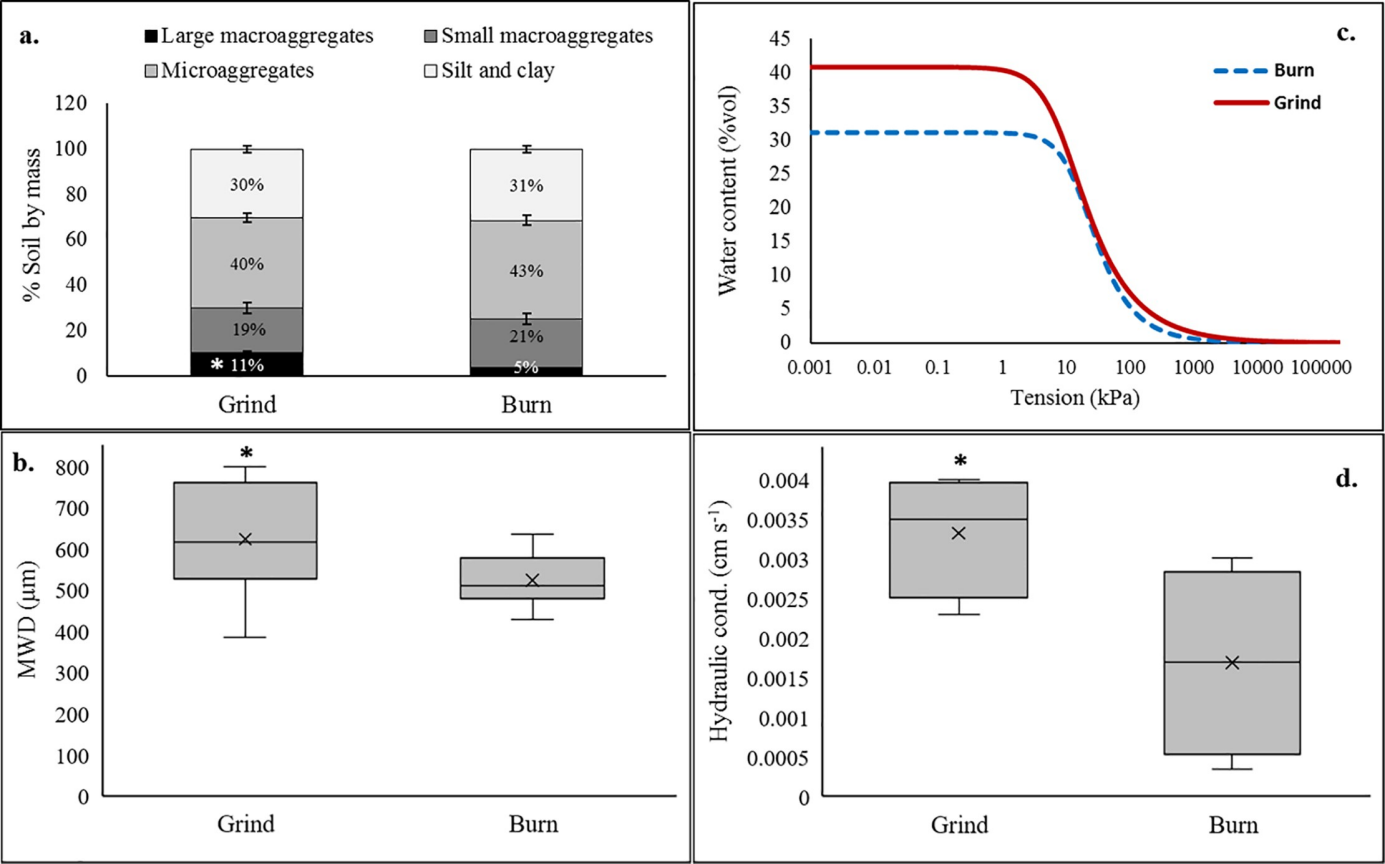
Effect of Grind and Burn treatments on (a) Proportion of soil aggregate fractions, (b) mean weight diameter (MWD), (c) water retention curves, and (d) infiltration rate. (*) indicate significant differences at *P ≤0*.*05*.

As the proportion of stable large macroaggregates increased (Fig 3A), WOR significantly enhanced aggregate stability (MWD) compared to the burn treatment (Fig 3B). Bulk density and compaction of topsoil layers (Table 1) were also significantly reduced under WOR (5 vs 16%). Water retention curves showed that the addition of woody biomass into the orchard soil had a significant effect on water retention in the topsoil. Soils were able to retain higher moisture (%VWC) at matric potential from 0 to ~10 kPA. The recycled treatment was able to store 30% more VWC at field capacity (FC) (Fig 3C). Water infiltration, measured as hydraulic conductivity (Fig 3D), was also significantly improved compared to the burn treatment (0.003 vs 0.001 cm s^-1^).

### Soil biology and SHI

**Fig 4:**
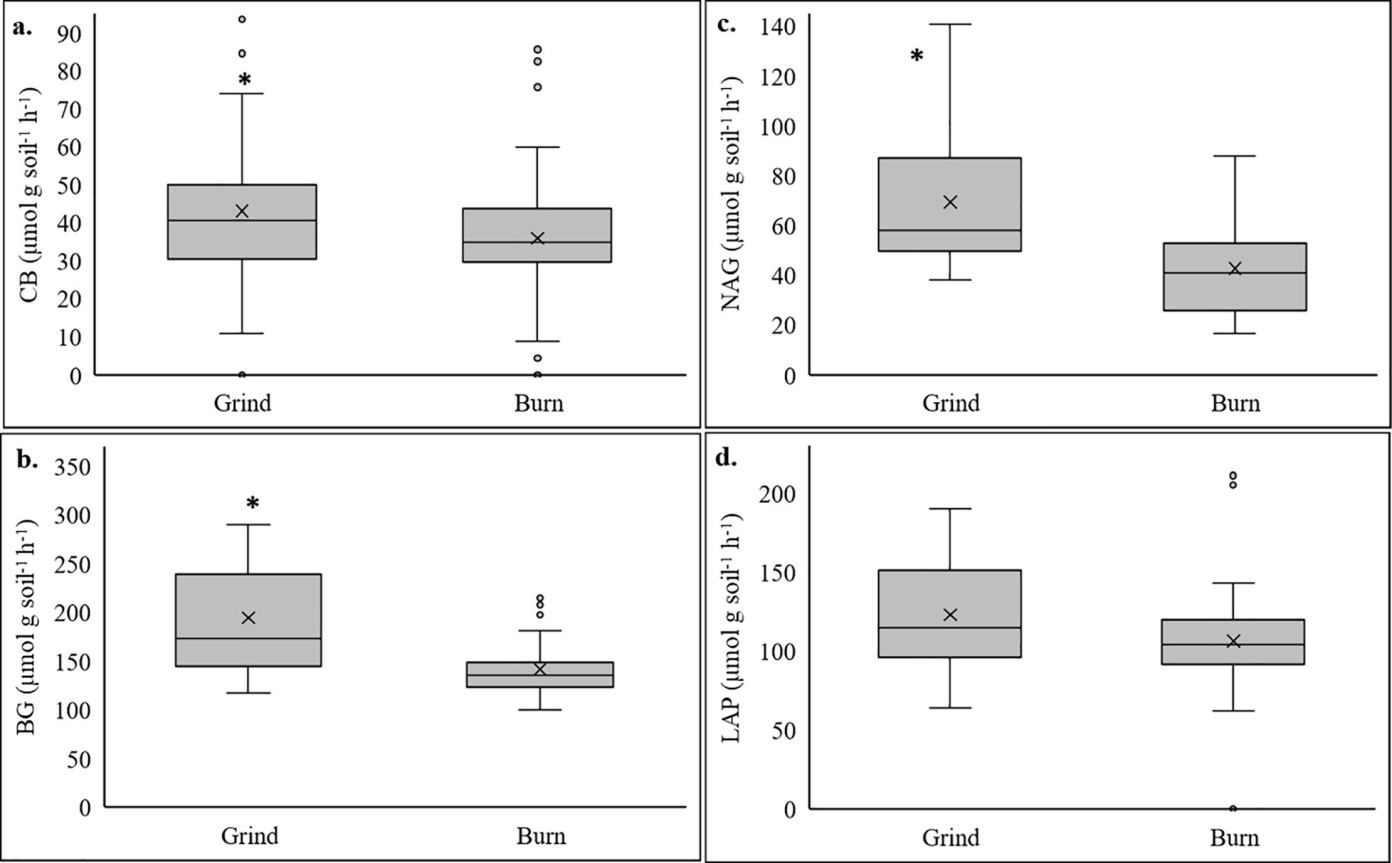
Effect of Grind and Burn treatments on (a) Cellulase (CB); (b) β -glucosidase (BG); (c) β-N-acetylglucosaminidase; (NAG), and (d) Leucine Aminopeptidase (LAP) enzyme activities. (*) indicate significant differences at *P ≤0*.*05*.

WOR significantly increased soil MBC by 28% compared to the burn (Table 2). While MBN trended higher (+13%), this increase was not significant (*P = 0*.*41*). Soil enzyme potential activity, which indicates potential rates of C and N cycling, was generally higher in the grind soil compared to the burn (Fig 4A–4D). The grind treatment soils had relatively higher levels of soil C cycling enzyme activity, with significant increases for both BG and CB (Fig 4A and 4B). Soil N cycling enzyme activity for NAG was also significantly increased under WOR, but not for LAP despite trending higher (Fig 4C and 4D). Additions of woody biomass into the orchard soil significantly increased estimated soil respiration rates (1-day CO_2_ evolution; +76%; *P ≤ 0*.*05*, Table 2). Because of these changes and shifts in WEOC, the soil health index (SHI) significantly increased from 4.24 in the burn treatment to 6.13 in the grind treatment (Table 2).

### Principal component analysis and correlations

**Fig 5:**
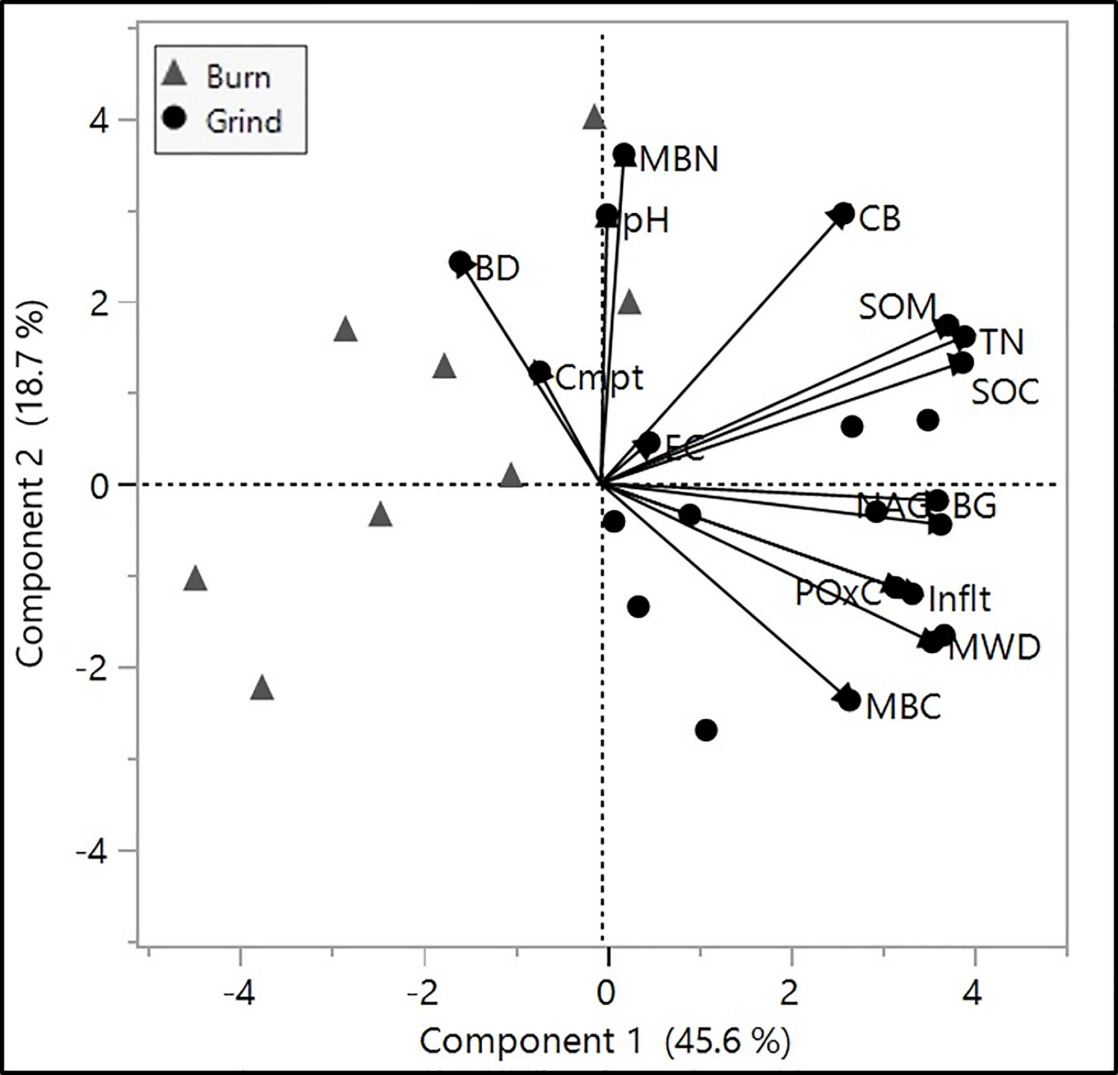
Principal Components Analysis (PCA) of soil health indicators and treatment clusters. BD, bulk density; Cmpt, compaction; Inflt, infiltration; MWD, mean weight diameter; EC, electrical conductivity; TN, total nitrogen; SOM, soil organic matter; SOC, soil organic carbon; POxC, permanganate oxidizable carbon; CB, cellulose; BG; β –glucosidase; NAG; β-N-acetylglucosaminidase.

Principal component (PCA) and correlation analysis were used to further identify relationships between soil properties across treatments (Fig 5, Table 3). We observed clear clustering of the two management practices and two principal components accounted for 64.3% of the total variance (PC1 and PC2 explaining 45.6% and 18.7% of total variance, respectively) (Fig 5). Soil parameters with high impact on treatment separation (>0.5) were soil C (SOC, SOM), total N, microbial biomass (MBC, MBN), soil enzymes (BG, CB, NAG) and physical properties such as aggregation (MWD), infiltration, and compaction. Total soil C (SOC) was strongly positively correlated with increases in total nitrogen, microbially stored C pools (MBC) and two enzymes (BG, NAG) (Table 3). Improvements in aggregation, infiltrations, soil water content and mitigation of compaction issues also trended with increases in SOC.

**Table 3:** Correlation coefficients among almond kernel yield, soil physicochemical, and biological properties as well as tree-water relations.

	Yield	MWD	Ift	Cpt	BD	TN	SOC	SOM	POxC	MBC	MBN	BG	CB	NAG	SWP	SCt	SWC
**Yield**	1.00																
**MWD**	0.62	1.00															
**Ift**	0.74*	0.60*	1.00														
**Cpt**	-0.17	-0.30	-0.61*	1.00													
**BD**	-0.90*	-0.59*	-0.83*	0.75*	1.00												
**TN**	0.75*	0.41	0.36	-0.40	-0.81*	1.00											
**SOC**	0.74*	0.43	0.51*	-0.43	-0.82*	0.99**	1.00										
**SOM**	0.83*	0.50*	0.64*	-0.40*	-0.87*	0.98**	0.98**	1.00									
**POxC**	0.46	0.54*	0.48	-0.15	-0.46	0.22	0.23	0.27	1.00								
**MBC**	0.38	0.66	0.76*	-0.07	-0.59	0.48	0.52*	0.52*	0.55*	1.00							
**MBN**	0.32	0.42	0.36	-0.15	-0.10	0.69*	0.15	0.19	0.49	0.57*	1.00						
**BG**	0.80*	0.64	0.44	-0.66*	-0.62*	0.68*	0.68*	0.74*	0.48	0.14	0.13	1.00					
**CB**	0.08	0.14	0.20	-0.32	-0.22	0.54*	0.53	0.44	0.18	0.18	0.62	0.31	1.00				
**NAG**	0.50	0.57	0.41	-0.75*	-0.51	0.72*	0.74*	0.68*	0.51	0.35	0.17	0.77*	0.50	1.00			
**SWP**	-0.56	-0.08	0.30	0.17	0.71	-0.16	-0.46	-0.75*	-0.06	-0.26	-0.58	-0.28	-0.56	-0.19	1.00		
**SCt**	0.43	0.66	0.22	-0.07	-0.25	0.24	0.27	0.21	0.73	0.64	0.45	0.12	0.18	0.52	-0.64*	1.00	
**SWC**	0.53*	0.72*	0.39	-0.27	-0.34	0.11	0.57*	0.55*	0.79	0.32	0.45	0.39	0.47	0.22	-0.30	0.59	1.00

*Significant at *P ≤ 0*.*05*

**Significant at *P ≤ 0*.*01*

Abbreviations: MWD, mean weight diameter; Ift, infiltration; Cpt; compaction; BD, bulk density; TN, total nitrogen; SOC, soil organic carbon; SOM, soil organic matter; POxC, Permanganate-oxidizable C, MBC, microbial biomass carbon; MBN, microbial biomass nitrogen; BG, β -glucosidase; CB, Cellulase; NAG, β-N-acetylglucosaminidase; SWP, stem water potential; SCt, stomatal conductance; SWC, soil water content.

### Resistance to deficit irrigation

**Fig 6:**
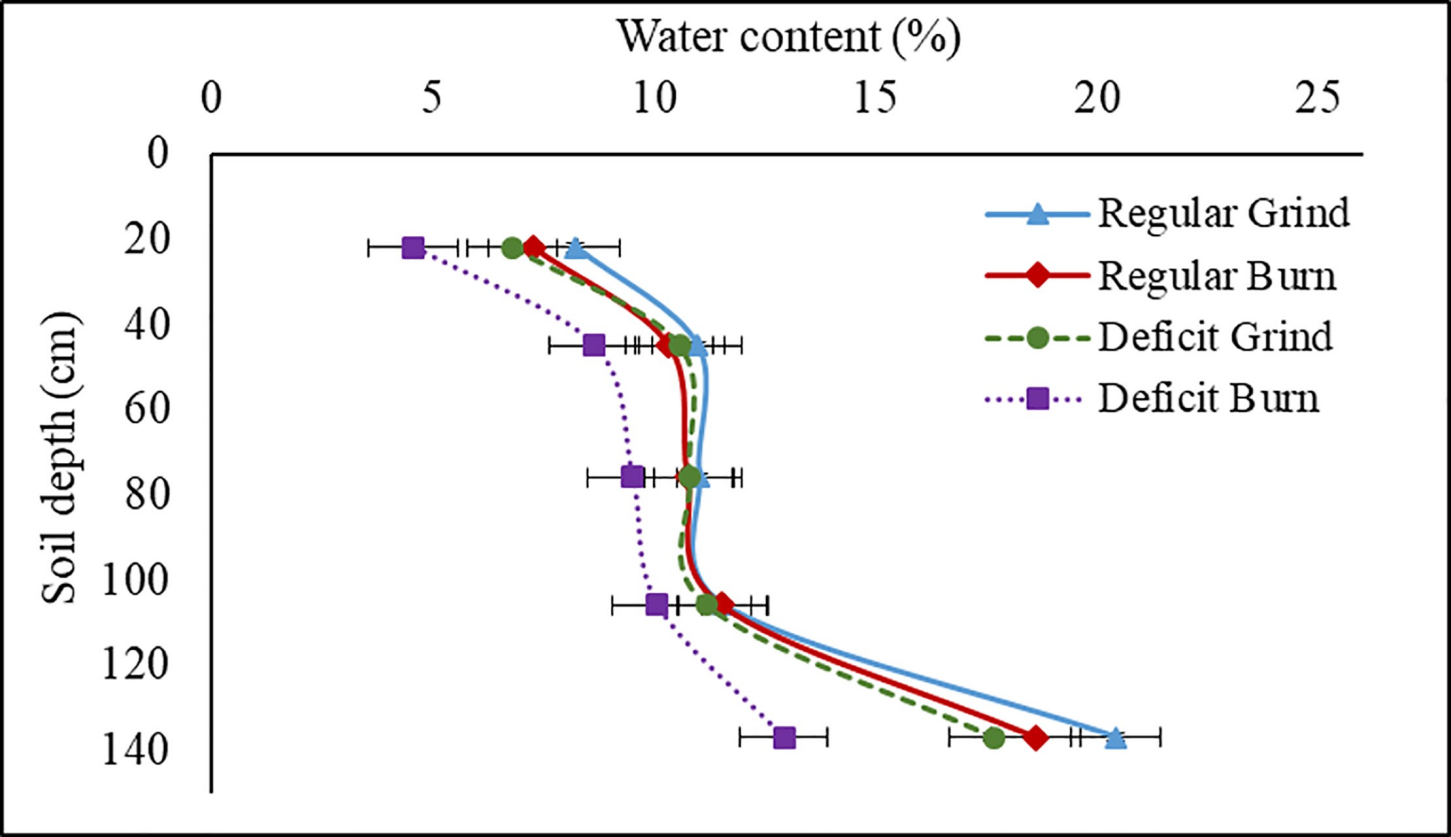
Average of neutron probe readings at different soil depths during the deficit irrigation period (6/5-7/3) as affected by soil (grind and burn) and irrigation treatments.

**Fig 7:**
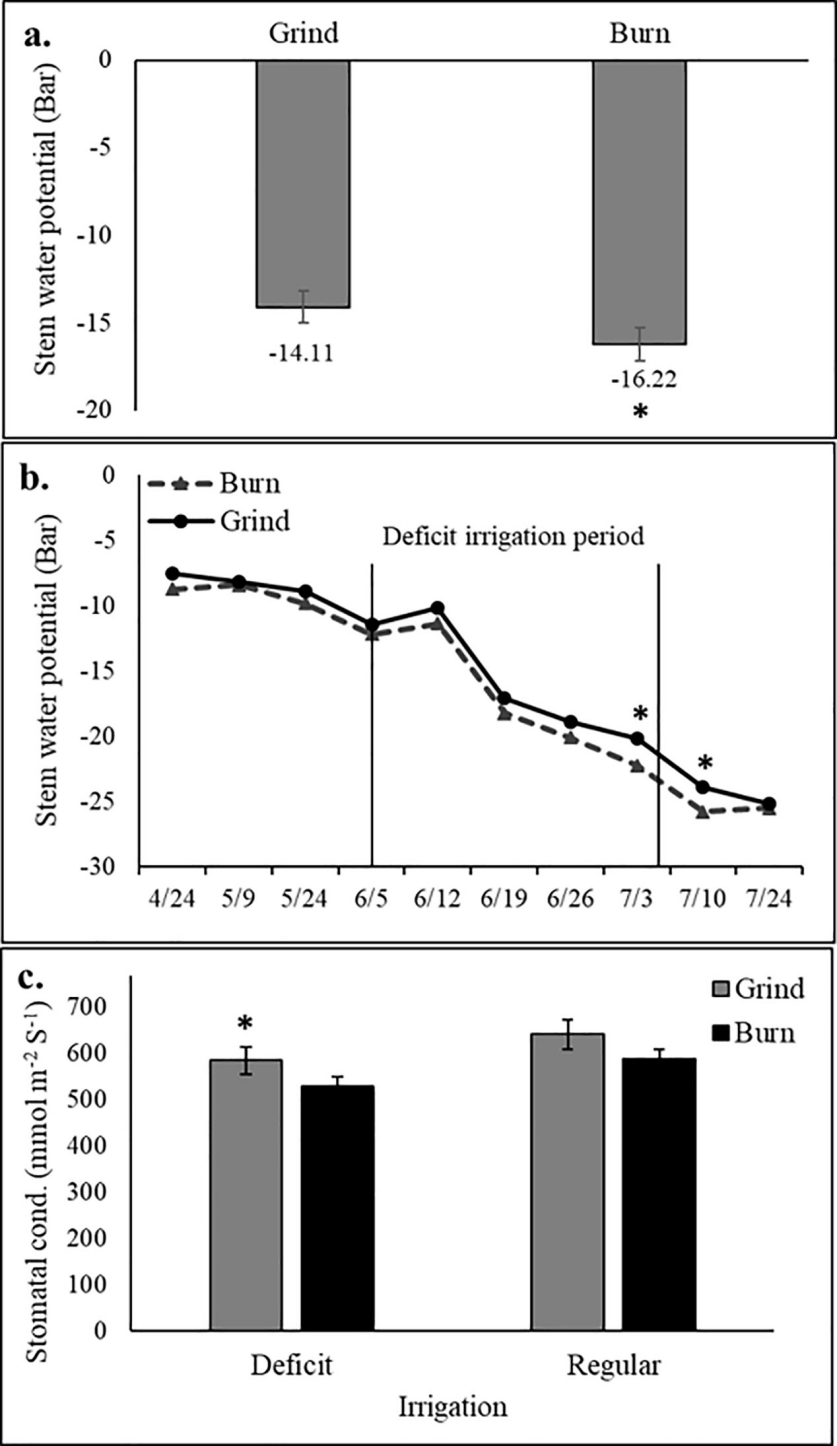
Effect of soil (Burn and Grind) and irrigation treatments on (a) average of weekly stem water potential readings (b) weekly measurements of stem water potential, and (c) stomatal conductance measurement (7/3). (*) indicate significant differences at *P ≤0*.*05*.

We tested the significance of changes in soil health parameters for tree and yield resistance to water shortages. Deficit irrigation resulted in a decrease in soil moisture in both treatments; however, the difference was more pronounced in the burn treatment (Fig 6). We observed a significant interaction between irrigation and soil treatments (*P = 0*.*05*), where the burn plots had a lower soil water content than the grind treatments under deficit irrigation, especially in the topsoil and at depth (Fig 6). We observed no significant interaction between soil treatment and irrigation on yields (*P = 0*.*25*, Fig 1B). However, stem water potential (SWP) measurements showed that trees in the grind plots maintained less negative value than those in the burn treatment (-14.11 vs -16.22 bar, respectively) over the course of the season (Fig 7A). Trees were significantly less water stressed on the last day of the deficit irrigation period in the grind treatment, as shown by significantly less negative SWP and higher stomatal conductance (Fig 7B and 7C). The grind treatment also assisted trees in their recovery post stress (Fig 7B). No changes in plant nutrition, canopy temperature and PAR interception were detected between treatments (S2 Table).

### Yields and soil health parameters

We examined correlations between soil health indicators and tree water status and yields (Table 3). Indicators of soil health improvement were positively correlated with almond yields. Significant positive correlations were observed between yield, soil N and C content (TN, SOC, and SOM) and C cycling (BG). Among soil physical properties, improvements in infiltration were significantly corrected with higher yields while bulk density and soil compaction trended negatively with yields. Total C content and SOM levels were also positively correlated with some orchard resiliency indicators (Table 3) such as soil water content and tree water status (SWP), where less negative SWP values indicate more favorable plant water status (Table 3).

## Discussion

The purpose of this experiment was to evaluate long term effects of WOR adoption on almond orchard functioning and climate change mitigation and adaptation services. We found that *in situ* biomass recycling prior to replanting orchards can help sequester a significant amount of C in the soil while improving yields and mitigating the impacts of acute water shortages on tree water stress. Although this practice can be costlier and challenging operationally on the short term, it offers a promising strategy for long term improvement in soil health and reduce risks of crop failure of orchard cropping systems.

### Biomass recycling provides substantial mitigation potential

The adoption of WOR is characterized by the reincorporation of large quantities of high C:N woody biomass (ratio of ~160) into orchard top soils. We found that the recycled orchard soils contained greater quantities of stored C compared to soils where residues were burned both when represented as a percentage (per 100g of soil) and total C stock per hectare in the topsoil (t ha^-1^). Taken over nine years, C storage services provided by this practice exceed the 4^o^/_oo_ targets set by the international community to significantly mitigate anthropogenic C concentration in the atmosphere [46–48].

WOR increased the formation and stability of large macroaggregates and preserved a larger quantity of intra-aggregate SOC relative to other aggregate fractions. The incorporation of woody biomass may provide sorption points for the formation of aggregate-scale microbial habitats while providing substrate for microbial growth, thereby providing energy and a favorable environment for particle aggregation and protection of soil C. A significant positive correlation between the SOM content, SOC, and aggregate MWD further confirms this trend, which was in accordance with the results reported by Fonte et al. [49], who found that macroaggregates were a large reservoir of organic C. A large input of plant residues in this system is likely one of the main factors underlying the improved content and stability of macroaggregates within the surface soil layer, resulting in more protection of soil C pools and longer turnover periods of sequestered C.

Labile carbon pools (POxC and MBC) also increased after biomass reincorporation. Differences in total SOC content of orchard soils were correlated to MBC, as well as BG and NAG activity potential, indicating that increased microbial biomass and C cycling under WOR had benefits for SOC storage. These results are in accordance with the observations of Bonanomi et al. [50], who found that high enzyme activities were mainly attributable to the relatively higher SOC and to energy sources and nutrients that sustain growth and activity of soil microbes. An increase in mineralization of organic substances from woody biomass decomposition may have in turn contributed to promoting the cementation of soil particles, forming macroaggregates and physically protected intra-aggregate SOC [51,52]. However, without any sustained C inputs, the C benefits of this practice might decrease over time, especially if smaller fast degrading woodchips are incorporated. Given the potential of this practice for C sequestration, holistic assessments of C turnover and the C footprint of WOR including emissions from added machinery is essential to more accurately predict the mitigation potential of this practice.

### Biomass recycling improves soil health metrics

In addition to contributing to achieving the long-term objective of limiting the global temperature increase, these changes in soil C were also crucial to improve soil fertility and functioning as a living ecosystem. Large additions of C as woody residues resulted in a higher soil health index and biological activity as represented by significant increases in microbial biomass, potential activity of soil enzymes, and microbial respiration. Decomposition of woody biomass releases substantial amounts of C into soil, where it is subsequently used to fuel microbial metabolism and cell production [53]. CB, an enzyme involved in cellulose hydrolysis, is essential in woody biomass decomposition. BG is a key member of the cellulase class of enzymes and completes the final step during cellulose hydrolysis by converting cellobiose to glucose [54]. Additions of woody biomass, composed of about 40% cellulose by weight, increased CB and BG potential activity under WOR. We found the same significant trend in the potential activity of NAG, involved in chitin degradation, that is likely due to an increase in fungal abundance [55]. These results suggest that incorporation of woody biomass has a notable positive effect on facilitating biologically active soils, capable of more effectively recycling and storing C and essential nutrients for plants and microbial processes.

Soil TN was also higher under WOR and was correlated to increases in microbial C cycling. The relatively high quantity of TN under WOR did not however translate to significant increases in inorganic N but did significantly correlate to increases in MBN. This indicates that competition for soil N may be higher under WOR and that, while more TN is present, a high quantity of it is microbially immobilized. The recycling of woody biomass could lead to increased N needs to sustain tree growth in the first year; however, it appears to increase the retention of N within the orchard system without affecting yields later on. Whether immobilized within microbial biomass, assimilated back into orchard biomass (as represented by significantly higher leaf N content and yield under WOR), or retained through binding to SOC and/or soil mineral surfaces, WOR likely decreases soil N leaching potential.

Biomass recycling also improved soil physical properties of importance to growers and efficient use of irrigation water. Soil compaction was alleviated, and it was negatively associated with improvements in SOC and BG enzymatic activity, indicating that microbial decomposition of woody inputs and subsequent enhancements in soil C may partially drive reductions in soil compaction. Increased volume of pore space resulting directly from chunky woody biomass additions to the topsoil might have also led to better porosity and reduction in compaction. Low soil surface compaction is an important component of fertile soils since it facilitates root growth, infiltration, and water and nutrient retention [56,57].

We observed a negative correlation between compaction metrics and soil water infiltration rates. Improvements in infiltration rates were likely attributed to an increase in soil porosity as shown previously [58] and increased preferential flow pathways and rates of hydraulic conductivity created after decomposition of large woody residues. Infiltration rate also positively correlated with aggregation, with macroaggregate stability understood as a key attribute impacting soil water movement [59]. In addition to infiltration rates, neutron probe readings and moisture retention curves indicated significant improvements in hydraulic properties. The 30% improvement in water retention at field capacity in the grind treatment could be in part attributed to increased formation and stabilization of soil aggregates associated with C additions and potential alteration of pore‐size distribution of amended soil [60]. These significant gains in soil water conservation could help better retain precipitation and water inputs, allowing growers to delay the onset or decrease the frequency of irrigation.

### Biomass recycling improves crop yield, productivity and water stress resistance

Trees following biomass recycling out-yielded those grown in the burn treatment under both irrigation scenarios. Yield benefits were significantly associated with differences in SOC between treatments, attributed to improvements in soil nutrition and moisture retention as well as increased soil biological activity [61,62]. Presumably, long term N supply from mineralization of SOM also contributed to higher yields in the grind plots. This was supported by greater accumulation of N in the leaves of grind trees compared to that of burn.

Biomass recycling decreased tree water stress as shown by a significantly less negative SWP throughout the course of the trial. The difference in SWP between the treatments was more pronounced when trees were at their most water stressed at hull split. Trees also showed greater recovery post stress after WOR. Stomatal conductance of WOR orchard trees was also significantly higher under the deficit irrigation treatment, and trended higher under regular irrigation. It has been shown that changes in SWP play a major role in short-term stomatal regulation of woody plants [63]. This was confirmed in our study, where a negative correlation was found between stomatal conductance and SWP. Trees under deficit irrigation conditions also decrease their stomatal conductance in response to a decrease in pre-dawn and mid-day leaf water potentials, in order to avoid substantial loss of moisture through transpiration [64,65]. WOR management has the potential to decrease water limitations of yields and increase tree endurance and yield resiliency potential in response to water shortages. These increases in yields coupled with reduced water stress make it an advantageous practice in semi-arid climates such as the Central Valley of California, where deficit irrigation is common pre-harvest and water scarcity is an increasingly serious challenge for growers.

## Conclusion

Despite added complexities and costs, we show that *in situ* biomass recycling holds great potential to sequester C in soils while improving yields and other ecosystem services that contribute to productivity and input use efficiency. Potential improvements in soil quality further highlight the beneficial implications of WOR for orchard adaptation to future climate variation and the potential of healthy soils to build resilience to irrigation supply shortages in almond orchards. The broad ecosystem services offered by orchard recycling make it a valuable alternative over the conventional methodology of in-field whole tree burning or hauling the tree biomass to the co-generation plants in California and elsewhere. Interdisciplinary evaluation of this practice regarding pest and disease potential and overall C footprint has shown no other major limitations [6], and overall C footprint assessments and best management practices are under development to realize the potential of this practice in a dynamic agricultural landscape.

## Supporting information

S1 Fig Almond kernel yield from 2014 to 2017 (a) as well as the cumulative kernel yields (b) at the Kearney site. Bars are standard error.
(TIF)

S1 File Spreadsheet data. (XLSX)

S1 Table Accumulation of mineral nutrients in the almond leaves as influenced by the irrigation and soil treatments. (DOCX)

S2 Table Photosynthetically Active Radiation (PAR), as well as yield per unit PAR intercepted, and canopy temperature in different soil and irrigation treatments. (DOCX)
