# Supplementary figures and images for: Orchard recycling improves climate change adaptation and mitigation potential of almond production systems
Source: PLoS One. 2020 Mar 27;15(3):e0229588. doi: 10.1371/journal.pone.0229588 (PMC7100960; doi:10.1371/journal.pone.0229588)

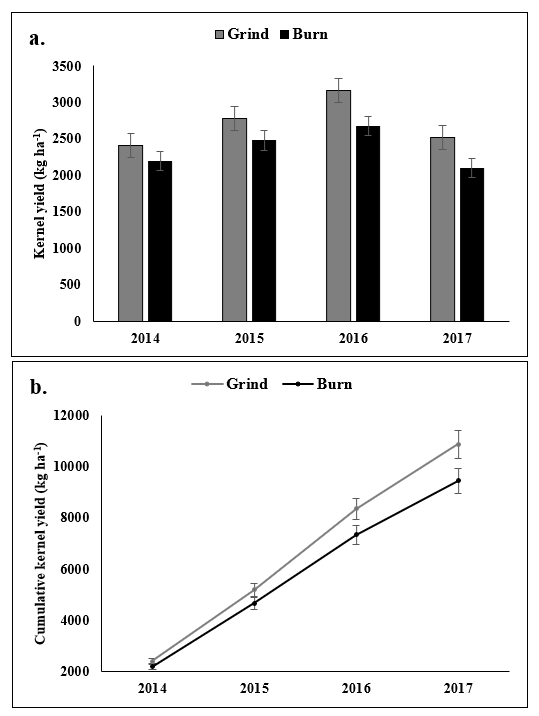

Supplement: S1 Fig — Almond kernel yield from 2014 to 2017 (a) as well as the cumulative kernel yields (b) at the Kearney site. Bars are standard error. (TIF) [file pone.0229588.s001.tif]
