# Supplementary material for: Orchard recycling improves climate change adaptation and mitigation potential of almond production systems
Source: PLoS One. 2020 Mar 27;15(3):e0229588. doi: 10.1371/journal.pone.0229588 (PMC7100960; doi:10.1371/journal.pone.0229588)
Supplement: S1 Table — (DOCX) [file pone.0229588.s003.docx]

**S1 Table. Accumulation of mineral nutrients in the almond leaves as influenced by the irrigation and soil treatments.**

| Irrigation | Treatment | N | P | K | Ca | Mg | Cl |
| --- | --- | --- | --- | --- | --- | --- | --- |
|  |  | …….……………….....…………. % DW^†^…….…….….……………………. | | | | | |
| Deficit | Grind | 2.56 | 0.12 | 1.31 | 3.85 | 1.07 | 0.12 |
|  | Burn | 2.48 | 0.12 | 1.26 | 4.05 | 1.09 | 0.11 |
| Regular | Grind | 2.55 | 0.11 | 1.33 | 3.91 | 1.09 | 0.11 |
|  | Burn | 2.50 | 0.12 | 1.29 | 4.06 | 1.05 | 0.10 |
| L.S^§^ | Irrigation | NS | NS | NS | NS | NS | NS |
|  | Treatment | * | NS | NS | NS | NS | NS |
|  | Interaction | NS | NS | NS | NS | NS | NS |
| Irrigation | Treatment | S | B | Zn | Mn | Fe | Na |
|  |  | ……………………….……… mg kg^-1^ DW…………….….…………………. | | | | | |
| Deficit | Grind | 1840 | 28.73 | 13.40 | 28.97 | 204 | 467 |
|  | Burn | 1786 | 27.30 | 12.57 | 24.60 | 228 | 361 |
| Regular | Grind | 1750 | 27.87 | 11.93 | 28.93 | 203 | 255 |
|  | Burn | 1733 | 28.40 | 13.20 | 28.00 | 206 | 350 |
| L.S | Irrigation | NS | NS | NS | NS | NS | NS |
|  | Treatment | NS | NS | NS | NS | NS | NS |
|  | Interaction | NS | NS | NS | NS | NS | NS |

^†^ Percent of leaf dry weight; ^§^ Level of significance; NS, not significant; * Significant at *P ≤ 0.05*
