# Supplementary material for: Orchard recycling improves climate change adaptation and mitigation potential of almond production systems
Source: PLoS One. 2020 Mar 27;15(3):e0229588. doi: 10.1371/journal.pone.0229588 (PMC7100960; doi:10.1371/journal.pone.0229588)
Supplement: S2 Table — (DOCX) [file pone.0229588.s004.docx]

**S2 Table. Photosynthetically active radiation (PAR), as well as yield per unit PAR intercepted, and canopy temperature in different soil and irrigation treatments.**

| Irrigation | Treatment | PAR (%) | Yield per unit PAR intercepted | Canopy temperature  (^o^ C) |
| --- | --- | --- | --- | --- |
| Deficit | Grind | 32.69 | 58.87 | 26.0 |
|  | Burn | 29.65 | 57.81 | 26.3 |
| Regular | Grind | 35.81 | 64.60 | 26.1 |
|  | Burn | 31.75 | 57.67 | 26.0 |
| L.S^†^ | Irrigation | NS | NS | NS |
|  | Treatment | NS | NS | NS |
|  | Interaction | NS | NS | NS |

^†^Level of significance; NS, not significant
